# Status of *Eimeria* Infection in Dairy Calves in and Around Bishoftu, Central Ethiopia

**DOI:** 10.1155/japr/8117528

**Published:** 2025-07-07

**Authors:** Yihenew Getahun Ambaw, Gelan Tafesa, Ambachew Motbaynor Wubaye, Simachew Getaneh Endalamew, Simegnew Adugna Kallu

**Affiliations:** ^1^College of Veterinary Medicine, Haramaya University, Dire Dawa, Ethiopia; ^2^Department of Veterinary Epidemiology and Public Health, School of Veterinary Medicine, Bahir Dar University, Bahir Dar, Ethiopia

**Keywords:** dairy calves, flotation, McMaster, prevalence of eimeriosis

## Abstract

Globally, eimeriosis is highly important for the health and productivity of calves. Although eimeriosis is one of the major causes of financial loss in cattle farming, there is inadequate evidence on the epidemiological status of infection in Ethiopia. Hence, this study was aimed at determining the prevalence and associated factors, assessing the burden, and identifying *Eimeria* species in dairy calves. A cross-sectional investigation of calf eimeriosis among dairy farms in Bishoftu, Central Ethiopia, was conducted from November 2023 to April 2024. A total of 384 calves aged from 3 weeks to 18 months were selected using simple random sampling, and fecal samples were collected rectally. Flotation technique was used to detect *Eimeria* oocysts, and the McMaster method was used to count OPGs. The prevalence of calf eimeriosis was 18.549% (95% CI: 14.90–22.72). For calves < 6 months (OR = 0.28, *p* < 0.001) and 12–18 months (OR = 0.31, *p* = 0.009), good body condition (OR = 0.23, *p* = 0.001), hygienic status (OR = 0.09, *p* < 0.001), and diarrheic calf status (OR = 3.24, *p* = 0.002) were significant (*p* < 0.05) factors for eimeriosis. The mean OPG score of the feces was significantly different among different age groups, fecal consistency groups, and housing systems. *Eimeria bovis* (28.17%), *Eimeria zuernii* (18.31%), and *Eimeria subspherica* (16.90%) were the most prevalent *Eimeria* species in this study. The prevalence of eimeriosis was high in calves; therefore, comprehensive calf management practices are essential to reduce the disease.

## 1. Introduction

Cattle population in Ethiopia is estimated to be 66 million and calves under 6 months account for 9% of the total. Among these, female cattle constitute 57% of the population and indigenous breeds represent 96.76%. The remaining breeds are cross (2.71%) and exotic (0.41%). Although Ethiopia has the largest livestock population in Africa, animal diseases are the main challenge in dairy production due to massive economic losses among dairy producers [1]. Among these diseases, diarrhea in calves is a severe form of syndrome that leads to significant loss of dairy production [2]. Calf diarrhea is caused by numerous infectious agents, such as bacteria, viruses, and protozoa [3]. Calf eimeriosis (synonym for coccidiosis) is one of the main protozoan diseases caused by *Eimeria* species and affects all calves found globally. The disease occurs most frequently and significantly in calves less than 1 year of age [3].

To date, more than 12 *Eimeria* species have been recognized in cattle. The major species of *Eimeria* are *Eimeria bovis*, *Eimeria zuernii*, *Eimeria auburnensis*, *Eimeria canadensis*, *Eimeria ellipsoidalis*, *Eimeria subspherica*, *Eimeria cylindrica*, *Eimeria alabamensis*, and *Eimeria wyomingensis* [4, 5]. However, the most prevalent species are *E. bovis* and *E. zuernii* [5]. Infectious diarrhea in calves can be influenced by many factors, such as the number of *Eimeria* oocysts present in the environment, calf frequency of exposure to oocysts, level of stressors in calves, *Eimeria* species interaction, environmental temperature, humidity, and sunlight [6]. Ingestion of sporulated oocysts in calves is the main method of infection for eimeriosis, which can spread quickly from calf to calf when animals are housed together or over congested and from dam to calf through unclean and polluted udders [7].

Although eimeriosis is an enteric disease that affects all ages of cattle, clinical eimeriosis is most common in calves. This phenomenon occurs primarily because of the incomplete development of immunity in young calves. Diseases are commonly encountered under intensive feeding conditions and management systems [8]. Calf eimeriosis frequently occurs in patients with subclinical disease with no apparent signs of infection. Eimeriosis causes injury to the intestinal lining of calves and results in reduced feed ingestion, malnourishment, poor growth, weight loss, and economic losses even though the animals appear healthy [9]. This disease causes significant economic losses in the meat and milk sector every year. In addition, it causes severe economic loss attributable to reduced appetite, weight loss, poor feed conversion rates, unthriftiness, diarrhea, dysentery, anemia, and treatment costs and increases susceptibility to further diseases [6]. This situation slows the age of the heifer at first calving, which again decreases the profit of the dairy sector [10].

*Eimeria* species are the main diarrhea-causing protozoan and intestinal pathogens affecting mainly calves and leading to serious calf illness and death in Ethiopia. Eimeriosis is the leading cause of economic loss in the livestock sector [11]. In cattle, eimeriosis is a communicable disease that leads to serious financial loss by diminishing body weight, resulting in a poor growth rate in animals and reduced production. As a result, the management approach for eimeriosis prevention and control in the livestock sector must be considered, particularly to identify the type of *Eimeria* species found on animal farms. Inappropriate control approaches for eimeriosis will increase the number of *Eimeria* cases owing to oocysts that remain to pollute the environment, which serve as feasible sources of transmission in animals. In cattle, sensitive diagnostic methods also play a significant role in prevention and control strategies for eimeriosis [12].

Ethiopia has a wide range of geographical and climatic conditions in numerous animal habitats; hence, understanding the epidemiological distribution of calf *Eimeria* species in several topographical setups is crucial. Most existing studies in the country have focused on adult animals. Although eimeriosis has a recognized impact on calf morbidity and mortality, there is a scarcity of up-to-date comprehensive evidence regarding the epidemiology of calf eimeriosis in Ethiopia. Therefore, this study was aimed at determining the prevalence of *Eimeria* infections and risk factors, identifying *Eimeria* species, and assessing the burden on dairy calves in Bishoftu, Central Ethiopia.

## 2. Materials and Methods

### 2.1. Study Area

**Figure 1:**
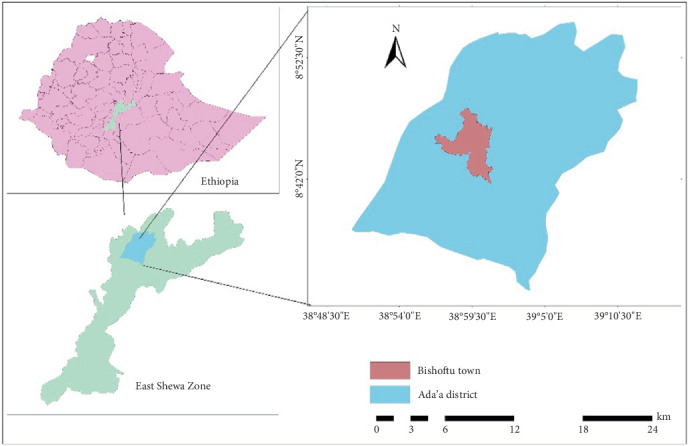
Map of the study area (Bishoftu town).

The investigation was carried out in Bishoftu town and its surroundings, which are located in Central Ethiopia (Figure 1). Geographically, Bishoftu is found in Ada'a district, far from 47 km to Addis Ababa in the eastern direction at 9°N latitude and 40°E longitude, at an elevation of 1850 m above sea level. The town has a subtropical highland climate with a bimodal rainfall distribution. The longer rainy season occurs from June to September, and the shorter rainy season occurs from March to May. The mean annual rainfall and relative humidity are 875 mm and 61.3%, respectively. The average annual temperature is 20°C, with a maximum of 26°C and a minimum of 14°C [13]. The study area (Bishoftu town) has a total livestock population of 98,586 cattle, 37,464 sheep, 17,481 goats, 52,023 donkeys, 7274 horses, and 535 mules [14].

### 2.2. Study Animals

The study population was all dairy calves within the age range of 3 weeks to 18 months, and calves more than 18 months and adults were excluded from the study. This age range was chosen because of the common occurrence of disease in young animals. Both the sexes and breeds (crossed and local) of dairy calves were the study participants. The sampled calves were categorized into three age groups: Group I (< 6 months), Group II (6–12 months), and Group III (12–18) [15], which were estimated by inquiring of the calf owners and records from the farms. The assessed calf body conditions were also divided into three groups: good, medium, and poor. This division is based on diverse body structures, visible bone parts, and fat deposits on calves [16].

### 2.3. Study Design

A cross-sectional study was carried out between November 2023 and April 2024 to determine the prevalence and determinants of coccidiosis in calves.

### 2.4. Sample Size Determination and Sampling Techniques

The minimum sample size required for this study was determined using the Thrusfield [17] formula. By considering the 95% confidence interval, 5% margin of error, and 50% expected prevalence, a total sample size of 384 was obtained:

 $$\begin{array}{c} N=\frac{1.96^{2}P_{\exp\left(1-p_{\exp}\right)}}{d^{2}} \end{array}$$

where *n* is the sample size, *z* (critical value) is 1.96 at a confidence level of 95% or at *α* = 0.05, *d* is absolute precision (0.05), and *p* is expected prevalence (50%).

A simple random sampling technique was employed to select both the study farms and individual calves on the farms in the study area.

### 2.5. Data Collection

Data were collected via the agrarians' record book and face-to-face interview with farmers on individual calf and farm-level information. Disposable plastic gloves were used to collect approximately 30 g of fresh fecal samples from each calf through the rectum. Throughout the sample collection, both farm- and calf-level factors, such as farm type, date of sampling, fecal consistency (diarrheic or nondiarrheic), age, sex, breed, and tag number, were documented for each calf using the prepared data extraction sheet. Potassium dichromate solution (2.5%) was added to the fecal sample placed in a labeled clean universal bottle. Afterwards, the sample was transported by keeping it in a cool ice box to Addis Ababa University, College of Veterinary Medicine, Veterinary Parasitology Laboratory, and preserved at refrigeration temperature until processing within 3 days of arrival.

### 2.6. Coprological Examination

The flotation method was employed with a concentrated sodium chloride solution with a specific gravity of 1.20 to examine the fecal samples [18]. To estimate the number of oocysts per gram (OPGs) of feces, 3 g of feces was taken from each calf fecal sample. Three grams of fecal samples and 42 mL of tap water were poured into a plastic beaker and mixed by stirring until the sample and water homogenized completely.

The homogenized mixture was sieved and centrifuged, and the sediment was resuspended in saturated Sheather's sugar solution. The sediment was painstakingly mixed with the saturated Sheather's sugar solution, filled into the chambers of a McMaster slide via a Pasteur pipette, and counted at 10x magnification via a microscope. The oocysts were enumerated to estimate the *Eimeria* load via the McMaster method as described by [19]. The samples that harbored *Eimeria* oocysts were mixed exhaustively with 2.5% potassium dichromate solution, allowed to sporulate for 2 weeks at room temperature [20]. In addition to sporulation, the fecal mixture was centrifuged, and the sediment was processed via the centrifugal flotation procedure with Sheather's sugar solution. The *Eimeria* species were identified on the basis of the morphology of the oocysts [21]. The size of the oocysts was measured via a calibrated micrometer under the 40x objective of a microscope. The microscopic morphology of *E. bovis* is ovoid and yellow in color and has a micropyle at the narrow end, whereas that of *E. zuernii* is spherical and colorless and does not contain a micropyle [22].

### 2.7. Statistical Analysis

The raw data were collected and recorded in a Microsoft Excel spreadsheet. Data management and analysis were performed in Stata Version 16 statistical tools. A chi-square test was performed to determine the associations between the presence of *Eimeria* and possible risk factors. Binary logistic regression was employed to examine the associations between predictor variables and the prevalence of *Eimeria*. Independent attributes with *p* values of less than 0.25 in the univariable analysis were incorporated into the multivariable model-building process. For the prevalence of eimeriosis, a forward stepwise multivariable binary logistic regression analysis was executed, starting from the attribute with the smallest *p* value in the univariable analyses. A *p* value of less than 0.05 in the multivariable binary logistic regression model was retained in the final model. The goodness of fit of the developed multivariable binary logistic regression model was assessed via the Hosmer–Lemeshow test for the calf dataset. The test statistic (*χ*^2^) was 11.50, with 7 degrees of freedom and a *p* value of 0.118, indicating that the given model fit the calf dataset. The mean and standard deviation were used to report the OPG of feces for each predictor variable. Independent *t*-tests and one-way ANOVA were also used to determine the differences between the mean OPGs of feces for various explanatory variables.

## 3. Results

### 3.1. Overall Prevalence of Eimeriosis

**Figure 2:**
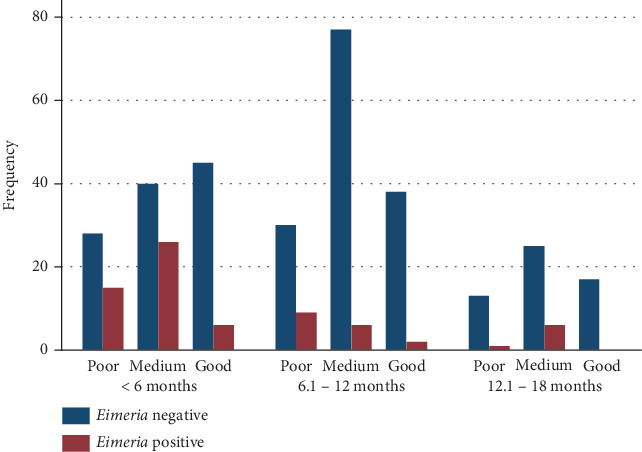
Status of eimeriosis by body condition and age group in Bishoftu, Ethiopia.

Three hundred eighty-four dairy calves were randomly selected to determine the prevalence and burden of eimeriosis by flotation and the McMaster method, respectively. Almost half of the study calves were female, 199 (51.82%), while 279 (72.66%) were crossbred (Table 1). Most calves were aged 6–12 months, 162 (162, 42.19%), followed by those aged less than 6 months, 160 (160, 41.67%) (Table 1). The current study revealed that among the 384 examined calves, 71 (18.49%) (95% CI: 14.90–22.72) were infected by *Eimeria*, whereas the remaining 313 (81.51%) (95% CI: 77.29–85.10) were free (Table 1). In terms of body condition, calves aged 6.1–12 months harbored more *Eimeria* than those aged 12.1–18 months with medium body weight conditions did (Figure 2).

The results of the chi-square test revealed that the variables age group, fecal consistency, body condition, farming system, and hygienic status of the farm were significantly associated (*p* value < 0.05) with the prevalence of eimeriosis in dairy calves, whereas sex, breed, and housing system were not (*p* value > 0.05) (Table 1).

### 3.2. Risk Factors for the Prevalence of Eimeriosis

The associations between the independent variables and the prevalence of eimeriosis were estimated via binary logistic regression analysis.  Table 2 displays the results of univariable and multivariable binary logistic regression analyses of eimeriosis infection.

For calves aged between 6 and 12 months, the odds of developing coccidiosis decreased 0.26-fold relative to those of calves aged less than 6 months (AOR = 0.26; 95%CI = 0.23, 0.53; *p* < 0.001). In calves 12–18 months of age, the odds of developing coccidiosis decreased 0.25-fold relative to those in calves < 6 months of age (AOR = 0.25; 95%CI = 0.09, 0.68; *p* = 0.007). In diarrheic calves, the odds of developing coccidiosis were 3.07-fold greater than those in nondiarrheic calves (AOR = 3.07; 95%CI = 1.52, 6.21; *p* = 0.002). For good body condition calves, the odds of developing coccidiosis were 0.22-fold lower than those for poor-conditioned calves (AOR = 0.22; 95%CI = 0.08, 0.59; *p* = 0.002). For calves in good hygiene farms, the odds of developing coccidiosis decreased by 92% relative to those calves in the poor hygiene farms (AOR = 0.08; 95%CI = 0.04, 0.15; *p* < 0.001) (Table 2).

### 3.3. Quantitative Fecal Examination

For quantitative fecal examination, the McMaster technique was used to count the number of OPG of feces. The minimum, mean, maximum, and standard deviation of the OPGs of the feces were 100, 3766.20, 12,800, and 3274.12, respectively. One-way ANOVA revealed that age group had a statistically significant (*p* value < 0.05) effect on the mean OPG score of *coccidian* oocysts in calves but not body condition (*p* value > 0.05). On the other hand, independent *t*-tests revealed that fecal consistency and housing system had statistically significant (*p* value < 0.05) differences in the mean OPG score of *coccidian* oocysts in calves but that of sex, breed, hygienic status of the farm, and farming systems were not significantly different (*p* value > 0.05) (Table 3).

### 3.4. Identification of *Eimeria* Species

**Figure 3:**
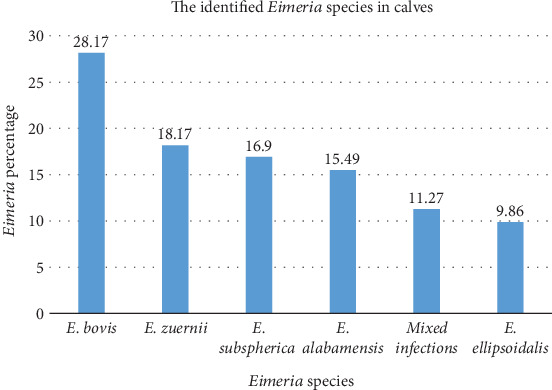
*Eimeria* species identified on the basis of oocyst morphology among dairy calves in Bishoftu, Ethiopia.

**Figure 4:**
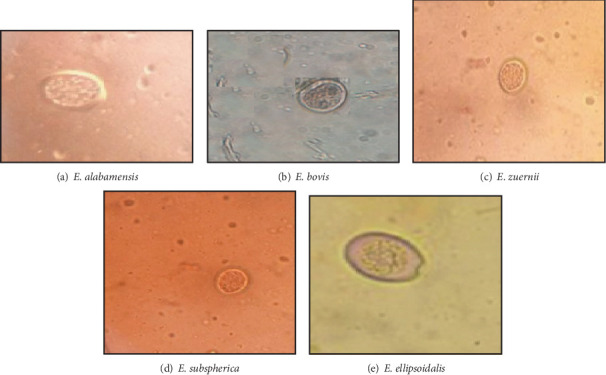
Photographs of *Eimeria* species among dairy calves in Bishoftu, Ethiopia.

In the present study, all dairy calves that harbor *Eimeria* (71) via flotation were further subjected to fecal culture to identify *Eimeria* species. Five *Eimeria* species were detected in this study. Among these, *E. bovis* and *E. zuernii* were the most dominant species (Figures 3 and 4).

## 4. Discussion

Globally, eimeriosis is a highly significant disease affecting bovine productivity and health [23]. Evidence on the epidemiology of this disease is crucial for disease management and the adoption of control programs [24]. In the present study, the prevalence of eimeriosis in calves was 18.49% (95% CI: 14.90–22.72), which is in line with previous reports in Ethiopia including 19.01% [25] and 20.10% [26] from Bahir Dar, 22.70% from Dire Dawa [27], 21.50% from southern Ethiopia [28], and 17.83% in Kacha Bira district [29].

This prevalence was lower than in other previous studies in Sebeta town (57.5%) [30], Thailand (35%) [31], Holeta (26.04%) [32], Kombolcha (31.90%) [33], Mekelle (72.70%) [34], Addis Ababa and Debre Zeit (68.10%) [35], Sekota (30.70%) [36], Bekoji (48.40%) [37], Addis Ababa (24.30%) [38], Jimma (31.00% [15] and 34.10% [39]), Bangladesh (55.60%) [23], and Indonesia (65.4%) [40]. This discrepancy in the prevalence of calf eimeriosis could be explained by the variation in different agricultural and farming practices used by the evaluated calves in various agroecosystems [41]. Geoecological inconsistency, along with various study designs, may explain the variation in the occurrence of disease in this study compared with other earlier studies. A certain study area may have high rainfall relative to others, and the majority of cattle in those areas may be managed in outdated farming systems where cattle graze pasture in swampy areas. This condition may lead to greater calf eimeriosis in Sebeta, Ethiopia [30], and in Bangladesh [23]. In addition, the variation in the prevalence estimate of this disease might be due to the presence of coexisting microbial infection, the amount of oocysts consumed by the calf, the climate situation, the season, the management conditions, the degree of immune status in the calf, and the types of diagnostic methods used in different reports [8].

In the present report, age group was a risk factor for calf eimeriosis, and calves from birth to 6 months of age were more likely to develop eimeriosis than calves from 6 to 18 months of age were. This result was comparable with other reports in Bahir Dar [26], Mekele [34], Dire Dawa [27], Jimma [39], Addis Ababa [38], Bekoji [37], Kacha Bira district [29], and Sekota [36]. However, this report was incomparable with the previous study of [15, 28, 30, 31, 33, 35]. The presence of eimeriosis in older calves (12–18 months) decreased by 78% relative to that in younger calves (< 6 months). This higher infection rate in younger calves could be due to a lack of farmer knowledge regarding colostrum feeding at the appropriate time to increase calf immunity [26]. The increased infection rate in younger calves may indicate that older calves develop higher levels of immunity that are influenced by prior exposure, as opposed to younger calves, which have no prior contact. Additionally, this demonstrated that younger calves' immune systems are still developing, making them more vulnerable to eimeriosis than older calves who have developed protection through prior exposure and are therefore more resilient to infections [42]. In the course of this examination, most calves younger than 6 months of age were kept in a congested environment, received less care, and were more likely to have contact with adult animals. This condition again promotes additional chances for calves to interact with each other and consumes more *Eimeria* oocysts, which leads to an increased risk of infection in young calves for Eimeriosis.

With respect to the hygienic status of the calf shed, calves living in an unclean house were more likely to develop eimeriosis than those living in a clean house. This result agrees with the report of [27, 36, 38, 43]. However, these findings are consistent with those of [15]. This high degree of eimeriosis infection in poor hygienic conditions could be due to inadequate housing management, unsanitary calving, and calf housing. Animal pastures with high concentrations also leave more excrement, which increases the chance of parasite eggs and oocytes contaminating the ground and endangering vulnerable calves [44].

The body condition of calves was significantly associated with eimeriosis infection. Poorly body conditioned calves had a greater risk of infection due to calf eimeriosis than calves with better body conditions. This result agrees with the previous report of [15, 23, 26, 32, 39]. On the other hand, this result disagrees with the findings of [33]. This may be because an animal's body condition has a direct bearing on its health and quality and is frequently cited as a key factor in determining its fitness [45]. Although all calves may have similar access to *Eimeria* oocysts, the greater prevalence of calf eimeriosis in calves with poor body condition than in those with poorer physical condition could be because those in better physical condition have greater immunity against infection. Furthermore, inadequate body conditions may result from inadequate feeding or nutritional supervision, which could impair calves' immune systems, reduce their resistance to infection, and increase the prevalence rate of animals in poor condition [41].

Fecal consistency was also a risk factor for the prevalence of eimeriosis. Compared with nondiarrheic calves, diarrheic calves were more likely to develop eimeriosis. This study agrees with the findings of [15, 28, 32, 36, 38]. This may be explained by each management system having a different approach to the hygienic system of the barn, nutritional status, feed contamination, and animal overcrowding. Moreover, there may be a connection between the increased susceptibility of cattle to *eimerial* infection and management factors. A greater number of risk factors for eimeriosis, including early weaning, failure to consume colostrum, and difficulty adjusting to artificial high-density diets, are present in calves raised in artificial environments [44].

In this study, the mean (3766.20 ± 3274.12) and maximum (12,800) levels of OPG in feces were relatively greater than those reported in and around Addis Ababa [38]. However, this value was lower than other studies in Addis Ababa and Debre Zeit [35]. The mean OPG level in calves was significantly greater in those with watery feces than in those with normal feces. This finding was in agreement with other authors [35, 38], which indicated that the occurrence of clinical infection is influenced by the amount of consumed oocysts. The study also demonstrated that the mean OPG level decreased with the age of the calves. The highest mean oocyst count was noted in calves < 6 months of age, followed by those aged 6–12 and 12–18 months. This greater amount of oocyst discharge in young calves could be due to the presence of an immature immune system in younger calves [42].

With respect to *Eimeria* species, we had identified five types of *Eimeria* species in this report in terms of the characteristics of calf oocysts as described by [19]. The current investigation revealed that the most frequently detected species were *E. bovis* (28.17%) and *E. zuernii* (18.31%), which is comparable with reports in Sekota, *E. bovis* (21.9%) and *E. zuernii* (12.5%) [36], and Bahir Dar, *E. bovis* (28.6%) and *E. zuernii* (19.0%) [26]. The finding was smaller than other previous studies of *E. bovis* (42.3%) and *E. zuernii* (28.3%) in Kombolcha [33] and greater than the reports of *E. bovis* (7.83%) and *E. zuernii* (3.25%) in Kacha Bira district and *E. bovis* (16.06%) and *E. zuernii* (7.40%) in Bangladesh [23]. These two *Eimeria* species are the most commonly reported eimeriosis outbreaks worldwide [42]. The proportions of single and mixed species infections caused by *Eimeria* species have been examined in infected calves. Among the 71 calves positive for *Eimeria* species, 63 (88.73%) and 8 (11.27%) were infected with single and mixed species, respectively. This difference in the detected species of *Eimeria* could be due to variation in the applied diagnostic test in the laboratory and agroclimate conditions [30].

## 5. Limitations of the Study

The study design employed was cross-sectional in nature; as a result, it cannot estimate the causal association of the prevalence of eimeriosis with the estimated risk factors. The applied laboratory technique (flotation) is not 100% sensitive; hence, the prevalence of eimeriosis may increase beyond this figure if we use advanced diagnostic tests such as PCR.

## 6. Conclusion

The current findings demonstrated that the prevalence and burden of eimeriosis were greater among calves in Bishoftu. Young calves, diarrheic fecal consistency, poor body condition, and poor hygienic status were significantly related to the prevalence of eimeriosis; however, sex, breed, housing, and farming system of the calves were not related to the prevalence of the disease. The mean OPG of feces was also significantly greater in young calves, diarrheal calves, and calves housed together with the mams. The greater mean amount of OPG in feces and the common occurrence of two extremely pathogenic species (*E. bovis and E. zuernii*) need special care in this regard. With respect to this report, focusing on the proper schedule of colostrum nourishing in younger calves and improving calf management practices among dairy farmers are encouraged to reduce the disease.

## Figures and Tables

**Table 1:** Chi-square analysis of factors associated with the prevalence of eimeriosis among dairy calves in Bishoftu, Ethiopia.

**Risk factors**	**Category**	**No. of examined**	**No. of positive**	**Prevalence (%)**	**χ** ^2^	**p** ** value**
Sex	Male	185	40	21.6	2.32	0.127
	Female	199	31	15.5		

Breed	Local	105	26	24.7	3.77	0.052
	Cross	279	45	16.1		

Age	< 6 months	162	47	29.01	20.61	< 0.001
	6.1–12 months	161	17	17.0		
	12.1–18 months	61	7	11.4		

Body condition	Poor	96	25	26.04	13.26	0.001
	Medium	180	38	21.1		
	Good	108	8	7.4		

Fecal consistency	Diarrheic	84	29	34.5	18.34	< 0.001
	Nondiarrheic	42	14	300		

Farming system	Intensive	268	42	15.1	19.02	< 0.001
	Extensive	116	29	25.0		

Housing system	Separate pen	223	37	16.5	1.27	0.260
	Together with cow	161	34	21.1		

Hygienic status of farm	Poor	129	54	41.8	70.40	< 0.001
	Good	255	17	6.6		

Overall prevalence	Positive	71	18.49 (14.90–22.72)			
	Negative	313	81.51 (77.29–85.10)	

Abbreviations: *χ*^2^, chi-square value; No., number.

**Table 2:** Univariable and multivariable binary logistic regression analysis of factors associated with the prevalence of eimeriosis among dairy calves in Bishoftu, Ethiopia.

**Risk factors**	**Category**	**cOR**	**95% CL**	**aOR**	**95% CI**	**p** ** value**
Sex	Male	Ref				
	Female	0.69	0.40, 1.12			

Breed	Local	1.71	0.99, 2.95			
	Cross	Ref				

Age	< 6 months	Ref				
	6–12 months	0.28	0.15, 0.52	0.25	0.12, 0.51	< 0.001
	12–18 months	0.31	0.13, 0.72	0.26	0.10, 0.72	0.009

Body condition	Poor	Ref				
	Medium	0.76	0.43, 1.36	0.67	0.33, 1.36	0.275
	Good	0.23	0.09, 0.53	0.20	0.08, 0.54	0.001

Fecal consistency	Diarrheic	3.24	1.86, 5.65	2.93	1.47, 5.82	0.002
	Nondiarrheic	Ref				

Farming system	Intensive	Ref				
	Extensive	1.75	1.02, 2.99			

Housing system	Separate pen	Ref				
	Together with cow	1.35	0.80, 2.26			

Hygienic status of farm	Poor	Ref				
	Good	0.09	0.05, 0.18	0.08	0.04, 0.16	< 0.001
Goodness of fit test	Hosmer–Lemeshow *χ*^2^ test with 7 *d*_f_ = 11.50					0.118

Abbreviations: aOR, adjusted odds ratio; CI, confidence interval; cOR, crude odds ratio; Ref, reference category.

**Table 3:** The mean oocyst counts among *Eimeria*-positive calves stratified by associated factors via independent *t*-tests and one-way ANOVA in Bishoftu, Ethiopia.

**Risk factors**	**Category**	**Mean**	**Standard error**	**[95% CI]**	**F**/**t-test**	**p** ** value**
Sex	Male	3406.25	536.82	2335.58, 4476.91	−1.05	0.296
	Female	4230.64	557.41	3118.92, 5342.36		

Breed	Local	4148.07	711.72	2728.59, 5567.56	−0.75	0.459
	Cross	3545.55	457.89	2632.30, 4458.80		

Age	< 6 months	4628.72	495.91	3639.65, 5617.78	5.47	0.006
	6–12 months	2307.14	843.49	624.85, 3989.43		
	12–18 months	1982.35	558.50	868.44, 3096.25		

Body condition	Poor	3938.00	636.27	2668.99, 5200.70	0.00	0.996
	Medium	3797.36	547.94	2704.52, 4890.21		
	Good	3506.25	1233.84	1045.43, 5967.06		

Fecal consistency	Diarrheic	5334.48	671.01	3996.18, 6672.77	−3.64	0.001
	Nondiarrheic	2683.33	391.69	1902.11, 3464.55		

Farming system	Extensive	3605.814	472.68	2663.08, 4548.54	−0.51	0.612
	Intensive	4012.5	674.62	2666.99, 5358.00		

Housing system	Separate pen	2655.405	446.63	1764.61, 3546.19	−3.17	0.002
	Together with cow	4975	589.07	3800.13, 6149.86		

Hygienic status of farm	Good	3468.519	408.31	2654.15, 4282.88	−1.37	0.174
	Poor	4711.765	964.89	2787.34, 6636.18		

Abbreviations: *F*, *F*-test; *t*, *t*-test.

## Data Availability

The data that support the findings of this study are available from the corresponding author upon reasonable request.
